# The Effect of a Nurse-Led Multidisciplinary Team on Ventilator-Associated Pneumonia Rates

**DOI:** 10.1155/2014/682621

**Published:** 2014-06-29

**Authors:** W. Bradley Dosher, Elena C. Loomis, Sherry L. Richardson, Jennifer A. Crowell, Richard D. Waltman, Lisa D. Miller, Muhammad Nazim, Faisal A. Khasawneh

**Affiliations:** ^1^School of Medicine, Texas Tech University Health Sciences Center, Amarillo, TX 79106, USA; ^2^Adult Critical Care Services, Northwest Texas Hospital, Amarillo, TX 79106, USA; ^3^Infection Control, Northwest Texas Hospital, Amarillo, TX 79106, USA; ^4^Respiratory Care, Northwest Texas Hospital, Amarillo, TX 79106, USA; ^5^Department of surgery, Texas Tech University Health Sciences Center, Amarillo, TX 79106, USA; ^6^Section of Critical Care Medicine, Department of Internal Medicine, Texas Tech University Health Sciences Center, 1400 S. Coulter Street, Amarillo, TX 79106, USA

## Abstract

*Background*. Ventilator-associated pneumonia (VAP) is a worrisome, yet potentially preventable threat in critically ill patients. Evidence-based clinical practices targeting the prevention of VAP have proven effective, but the most optimal methods to ensure consistent implementation and compliance remain unknown. *Methods*. A retrospective study of the trend in VAP rates in a community-hospital's open medical intensive care unit (MICU) after the enactment of a nurse-led VAP prevention team. The period of the study was between April 1, 2009, and September 30, 2012. The team rounded on mechanically ventilated patients every Tuesday and Thursday. They ensured adherence to the evidence-based VAP prevention. A separate and independent infection control team monitored VAP rates. *Results*. Across the study period, mean VAP rate was 3.20/1000 ventilator days ±5.71 SD. Throughout the study time frame, there was an average monthly reduction in VAP rate of 0.27/1000 ventilator days, *P* < 0.001 (CI: −0.40–−0.13). *Conclusion*. A nurse-led interdisciplinary team dedicated to VAP prevention rounding twice a week to ensure adherence with a VAP prevention bundle lowered VAP rates in a community-hospital open MICU. The team had interdepartmental and administrative support and addressed any deficiencies in the VAP prevention bundle components actively.

## 1. Introduction

Ventilator-associated pneumonia (VAP) is a common device-related infection. Its incidence ranges from 8% to 28% and it increases with the duration of mechanical ventilation (MV) [1]. It has multiple negative impacts on patients' care, including prolonged intensive care unit (ICU) stay, excess healthcare costs, and higher mortality [2–5]. In lights of all the ill-desired consequences mentioned above, VAP prevention has been proposed as a measure of quality-of-care [4].

Shortly after hospital admission, the upper airways become colonized with a variety of potentially virulent bacteria, some of which can be multidrug resistant like methicillin-resistant* Staphylococcus aureus *and* Pseudomonas aeruginosa* [1, 4, 6]. Endotracheal intubation interferes with the protective mucociliary action in the airways and it renders the cough reflex less effective [1, 4, 6]. This facilitates the aspiration of colonized nasopharyngeal secretions from around and within the endotracheal tube leading to lower airways' colonization and subsequent VAP.

Different studies, and, subsequently, professional guidelines, have suggested a group of measures to block the above cascade of events and ultimately prevent VAP [7–10]. These measures include keeping the head of bed elevated at 30–45 degrees, performing regular oral care with chlorhexidine antiseptic solution, suctioning subglottic secretions regularly, using gastrointestinal and deep venous thrombosis prophylaxis, daily spontaneous breathing trials when the patient is eligible, and holding sedation at least once daily.

Despite of the multitude of studies addressing the issue of VAP prevention, a number of significant methodological flaws existed which limits the ability to establish the effectiveness of different prevention measures [11]. Furthermore, the most optimal method to ensure the consistent implementation of these measures remains unknown.

This retrospective study examined the effect of a nurse-led VAP prevention team on VAP rates in an open medical ICU (MICU) in a community-hospital.

## 2. Methods

This retrospective study was performed at Northwest Texas Hospital (NWTH), a 400-bed community-hospital in Amarillo, Texas, with an open 18-bed MICU. NWTH is one of the teaching hospitals for Texas Tech University Health Sciences Center (TTUHSC) in Amarillo, Texas. It is a trauma center that serves about half a million inhabitants in the Texas panhandle. In the MICU, a board-certified intensivist leads daily multidisciplinary morning rounds on the teaching service, which comprises about half of the MICU patients. The intensivist is available after hours by phone or in-person, if needed. Hospitalists and private physicians are mandated to consult an intensivist if they admit a mechanically ventilated patient; otherwise they consult on as needed bases. The nurse to patient ratio in the MICU rarely exceeds 1 : 2 and about 25% of the MICU nurses are certified critical care nurses (CCRN). The institutional review boards of both TTUHSC and NWTH approved this study protocol.

The study period extended from April 1, 2009, until September 30, 2012. The VAP prevention team consisted of the MICU nursing director, MICU nursing educator, a dedicated respiratory therapist, and an infection control nurse. The team rounded on mechanically ventilated patients every Tuesday and Thursday. Using a standard check list, they ensured adherence with the evidence-based VAP prevention bundle: head-of-bed elevation to 30–45 degrees, daily sedation vacation, daily spontaneous breathing trials if feasible, regular chlorhexidine oral care, regular suctioning of subglottic secretions, use of deep venous thrombosis prophylaxis, and gastrointestinal prophylaxis. Missing VAP bundle elements were rectified as soon as possible by contacting the patient's treating physician. The intention was to achieve 100% compliance with the above bundle. The MICU nurse educator provided constant bedside teaching to floor staff throughout the study period. Suggestions based on the VAP prevention team's round findings were conveyed quarterly by the MICU nursing director to the appropriate hospital committees: Infection Prevention Committee, Critical Care Committee and Hospital Performance Improvement Committee. A separate and independent infection control team monitored VAP rates. The team consisted of 2 infection preventionists (nurses with interest and training in infection control) who have not changed during the study period. The above-mentioned team used to review tracheal aspirate cultures sent by the microbiology lab on all MICU patients daily. The charts of patients with positive culture results were reviewed further for clinical and radiological data supporting VAP diagnosis. The infection control team was aware of the VAP prevention team efforts.

VAP was diagnosed if an intubated patient with positive airway cultures met the following criteria:

- new or progressive and persistent pulmonary opacities on chest radiographs;
- at least one of the following: fever or hypothermia with no other recognized cause, leukopenia, or leukocytosis;
- at least two of the following: new onset purulent airway secretions or change in the amount or character of airway secretions, rales or bronchial breath sounds on physical examination, or worsening gas exchange.

All of the above VAP prevention measures were implemented from the outset of the study and no new measures were introduced during the study time frame.

In the MICU, a respiratory therapist checks mechanical ventilators every 4 hours. He documents ventilator parameters, weans oxygen down, gives nebulizers, suctions airways, if needed, and suctions the subglottic secretions. Education and regular reminders were the methods used to encourage respiratory therapist's adherence with the above intervention with no other measure used to ensure compliance. Through the study period the bedside nurse provided oral care every 6 hours with 0.12% chlorhexidine swabs used every 12 hours.

Continuous data were expressed as a mean with its associated standard deviation (SD). Categorical data was presented as number of variables with corresponding percentages. Time series regression analysis was used to test the statistical significance of the trend in data. Student's *t*-test was used to compare means. The statistical analysis was done using Stata 12 (StataCorp LP, College Station, Texas). A *P* value < 0.05 was considered statistically significant.

## 3. Results

During the study period 4383 patients were admitted to the MICU. Average length of stay was 3.44 ± 0.49 days. The mean Acute Physiology and Chronic Health Evaluation (APACHE) II score was 12.86 ± 0.88 and the mean ICU mortality was 6.51% ±1.58. Neither variable showed a significant trend throughout the study period, and both were similar to their previous values before initiating the study intervention. During the study period the VAP team rounded on 713 (49.0% males) out of 1148 mechanically ventilated patients (62.1%). The mean age of this cohort was 58.8 years ±17.5 SD. The mean duration of mechanical ventilation during the study period was 72.3 hours ±65.3 SD in 2009, 89.1 ± 79.7 SD hours in 2010, 60.8 hours ±62.2 SD during 2011, and 68.7 hours ±65.1 SD during 2012. There was no significant trend noted in the duration of mechanical ventilation during the study period. A checklist was used and filled out prospectively as the VAP prevention team rounded on mechanical ventilated patients. It included demographic variables and the interventions to be implemented. The VAP prevention team did not collect any specific data about the other 435 patients (37.9%).

In our hospital, endotracheal tubes that allow subglottic secretions suctioning are stocked in the emergency department, operating room, and MICU. During the study period, 121 patients (17.0%) did not have the above-specialized tubes used. They were intubated in the field or transferred from other facilities and they were managed without changing their endotracheal tubes unless indicated.

Across the study period VAP rates had a minimum value of 0.00/1000 ventilator days and maximum value of 17.24/1000 ventilator days. Mean VAP rate was 3.20/1000 ventilator days ±5.71 SD. In the two quarters before the study began, the mean VAP rate was 9.32/1000 ventilator days ±7.38 SD, which was similar to the rates in the first 2 quarters of the study (*P* = 0.28).

**Figure 1:**
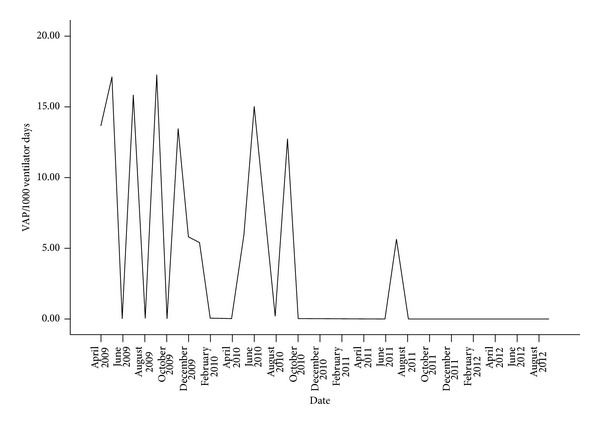
Time series plot of the ventilator-associated pneumonia rate from April 2009 to September 2012.

Along the course of the intervention, the VAP prevention team identified multiple areas of improvement: new order sets to ensure adherence with the above VAP prevention bundle were put forward, knowledge gaps were addressed at the bedside as well as in the class room for the incoming nurses and resident physicians, feedback regarding the change in VAP rates was provided to the hospital's Critical Care Committee and treating physicians, and interdepartmental obstacles were dealt with through communication with hospital administration and appropriate hospital committees. Each member of the VAP prevention team promoted adherence with the VAP prevention bundle by talking to their colleagues and the attending physicians using the MICU services. Throughout the study time frame, there was an average monthly reduction in VAP rates of 0.27/1000 ventilator days, *P* < 0.001 (CI: −0.40–−0.13); see Figure 1 and Table 1.

## 4. Discussion

In this study, adherence with a VAP prevention bundle was associated with a significant decrease in the incidence of VAP. A dedicated team consisting of the MICU nursing director, MICU nursing educator, infection preventionist, and a respiratory therapist rounded twice weekly on mechanically ventilated patients to ensure adherence with a VAP prevention bundle. The team rounded in the early afternoon after formal MICU rounds ended. The team targeted full adherence with the prevention bundle and addressed any deficiencies on the spot. Over the study period, the team was able to achieve a significant reduction in VAP rates. The above success hinged on education, consistent monitoring to change behavior, recruiting interdisciplinary collaboration, and summoning the support of all the stakeholders.

In a study by Mendez et al., a ventilator bundle rounding team consisting of an attending physician (not the rounding attending physician), nursing leadership, respiratory therapist, and a pharmacist rounded on mechanically ventilated patients on weekdays [12]. The team promoted adherence with the VAP prevention bundle. This improved compliance with nurse-driven sedation vacation, but not physician-driven peptic ulcer disease prophylaxis or respiratory therapist-driven spontaneous breathing trials.

Our study differed from the above study in multiple aspects; our VAP prevention team did not include a physician or a pharmacist which would facilitate rounding-time scheduling and the team rounded only twice a week which made the efforts more sustainable and avoided burn out. Although physicians were not involved in the actual rounding, they were kept informed of progress and their input was solicited at multiple occasions.

In a single center 2-year pre- and postintervention observational study, the effect of compliance with eight preventive measures on VAP rates was examined [13]. The interventions implemented were hand hygiene and glove-and-gown use, head of bed elevation to 30–45 degrees, maintaining endotracheal tube cuff pressure at no less than 20 cm of H_2_O, favoring orogastric tubes over nasogastric tubes, avoiding gastric overdistension during tube feeding, good oral hygiene with chlorhexidine, and eliminating nonessential tracheal suctioning. A multidisciplinary task force consisting of 4 intensivists and 1 infection control physician overlooked the process. The implementation was facilitated through educational sessions, direct observations with performance feedback, technical improvements, and reminders. Compliance with the above measures increased steadily over time and was associated with a 51% decrease in VAP prevalence rate.

Although our data has similarities with the efforts above, it evaluated the contribution of a different set of interventions, which included daily sedation vacation, daily spontaneous breathing trial, and subglottic secretions suctioning, on VAP rates. Furthermore, physicians contributed to the preventive efforts of VAP but were not the driving force behind it.

Reviewing the above studies and others evaluating VAP prevention stresses out the fact that compliance with VAP prevention bundles and tools used to improve its enactment varies between institutions [7, 10, 14–16]. Moreover, dedicated VAP prevention teams, like the one we assembled, have proven effective but the optimal makeup of the team members and the ideal rounding schedule still needs further investigation.

Aspiration of heavily colonized upper airway secretions is a pivotal step in the pathogenesis of VAP. The cuffs of endotracheal tubes are effective in preventing macroscopic but not microscopic aspiration. A meta-analysis of 13 randomized clinical trials comprising of more than 2400 patients demonstrated a 50% reduction in VAP rates among patients receiving continuous or intermittent subglottic secretions suctioning [17]. It also showed a reduction in ICU LOS and duration of mechanical ventilation. Furthermore, when VAP occurred, it was diagnosed at a later time compared with patients without this intervention. Moreover, the number needed to be treated with this technology to prevent one case of VAP was 11 which justifies the cost of these endotracheal tubes in light of the higher cost associated with treating VAP.

## 5. Limitations

This study has limitations; first it represents a single center experience that needs to be corroborated by others. Second, we did not document compliance rates with different VAP prevention bundle components prior to initiating this intervention. During the intervention full compliance with nurse-driven, physician-driven, and respiratory therapist-driven components of the bundle was the goal. Any deficiency in implementation would have generated a phone call to the treating physician to give a verbal order addressing the missing component. Third, we did not gather data to examine the effect of VAP rates reduction on clinically important patient outcomes like antimicrobial utilization or ICU LOS or mortality in mechanically ventilated patients. Moreover, we believe that sending patients whom we expect to need lengthy mechanical ventilation to long-term acute care hospitals biased the duration of mechanical ventilation in our cohort. Hence, it did not show a favorable trend with the drop in VAP rates. Lastly, we utilized the Centers for Disease Control and Prevention VAP definition despite of its limited accuracy and interobserver reliability [16, 18–20]. Our infection prevention team was consistent throughout the study period and was systematic in their data collection, which should have helped in mitigating the shortcoming of the above-mentioned definition.

## 6. Conclusion

The VAP prevention bundle is effective and compliance with its components lowers VAP rates. In this study, a nurse-led VAP prevention team rounding twice weekly was able to significantly lower VAP rates in a community-hospital's open MICU. The intervention revolved around multidisciplinary participation and bedside and classroom education, in addition to support from all stakeholders—including hospital administration and physicians. Nevertheless, there continues to be an obvious need for more research to identify other effective methods that increase adherence and ensure consistent implementation of the above-mentioned bundle.

## Figures and Tables

**Table 1:** Monthly ventilator-associated pneumonia rate from April 2009 to September 2012.

Month	Pneumonia MICU	Vent. days	MICU
April 2009	2	147	13.605
May 2009	2	118	16.949
June 2009	0	112	0
July 2009	2	126	15.873
August 2009	0	116	0
September 2009	3	174	17.241
October 2009	0	154	0
November 2009	3	222	13.514
December 2009	1	173	5.78
January 2010	1	185	5.405
February 2010	0	182	0
March 2010	0	156	0
April 2010	0	140	0
May 2010	1	186	5.376
June 2010	2	136	14.706
July 2010	1	129	7.752
August 2010	0	150	0
September 2010	2	156	12.821
October 2010	0	155	0
November 2010	0	88	0
December 2010	0	137	0
January 2011	0	115	0
February 2011	0	224	0
March 2011	0	151	0
April 2011	0	151	0
May 2011	0	218	0
June 2011	0	116	0
July 2011	1	184	5.435
August 2011	0	78	0
September 2011	0	131	0
October 2011	0	185	0
November 2011	0	128	0
December 2011	0	110	0
January 2012	0	124	0
February 2012	0	142	0
March 2012	0	183	0
April 2012	0	167	0
May 2012	0	85	0
June 2012	0	98	0
July 2012	0	105	0
August 2012	0	105	0
September 2012	0	128	0
